# Putative Sugar Transporters of the Mustard Leaf Beetle *Phaedon cochleariae*: Their Phylogeny and Role for Nutrient Supply in Larval Defensive Glands

**DOI:** 10.1371/journal.pone.0084461

**Published:** 2013-12-31

**Authors:** Magdalena Stock, René R. Gretscher, Marco Groth, Simone Eiserloh, Wilhelm Boland, Antje Burse

**Affiliations:** 1 Department of Bioorganic Chemistry, Max Planck Institute for Chemical Ecology, Jena, Thuringia, Germany; 2 Genome Analysis Group, Leibniz Institute for Age Research – Fritz Lipmann Institute, Jena, Thuringia, Germany; CNRS, France

## Abstract

**Background:**

Phytophagous insects have emerged successfully on the planet also because of the development of diverse and often astonishing defensive strategies against their enemies. The larvae of the mustard leaf beetle *Phaedon cochleariae*, for example, secrete deterrents from specialized defensive glands on their back. The secretion process involves ATP-binding cassette transporters. Therefore, sugar as one of the major energy sources to fuel the ATP synthesis for the cellular metabolism and transport processes, has to be present in the defensive glands. However, the role of sugar transporters for the production of defensive secretions was not addressed until now.

**Results:**

To identify sugar transporters in *P. cochleariae*, a transcript catalogue was created by Illumina sequencing of cDNA libraries. A total of 68,667 transcripts were identified and 68 proteins were annotated as either members of the solute carrier 2 (SLC2) family or trehalose transporters. Phylogenetic analyses revealed an extension of the mammalian GLUT6/8 class in insects as well as one group of transporters exhibiting distinctive conserved motifs only present in the insect order Coleoptera. RNA-seq data of samples derived from the defensive glands revealed six transcripts encoding sugar transporters with more than 3,000 counts. Two of them are exclusively expressed in the glandular tissue. Reduction in secretions production was accomplished by silencing two of four selected transporters. RNA-seq experiments of transporter-silenced larvae showed the down-regulation of the silenced transporter but concurrently the up-regulation of other SLC2 transporters suggesting an adaptive system to maintain sugar homeostasis in the defensive glands.

**Conclusion:**

We provide the first comprehensive phylogenetic study of the SLC2 family in a phytophagous beetle species. RNAi and RNA-seq experiments underline the importance of SLC2 transporters in defensive glands to achieve a chemical defense for successful competitive interaction in natural ecosystems.

## Introduction

Sugar sweetens our life. Glucose is one of the major energy sources and an important substrate for both protein and lipid synthesis. Its catabolism fuels cellular respiration for ATP production. For glucose absorption in the mammalian small intestine two pathways are known: the passive, paracellular absorption which bats rely on for more than 70% of their total sugar uptake [1] and the transporter-mediated transcellular pathways which non-flying mammals use preferentially. They take up glucose from interstitial fluid by a passive, facilitative transport process driven by the downward concentration gradient across the plasma membrane [2]. Exclusively in the epithelial cell brush border of the small intestine and the kidney proximal convoluted tubules, glucose is absorbed or reabsorbed against its electrochemical gradient by a secondary active transport mechanism which uses the sodium concentration gradient established by Na^+^/K^+^/ATP pumps [3]. Unlike glucose in mammals, the major blood (hemolymph) sugar of insects is often the disaccharide trehalose (α-D-glucopyranosyl-α-D-glucopyranoside) [4]–[6]. It is synthesized from glucose phosphates in fat body tissue and serves as a source of carbohydrates for various tissues including flight muscles [7], [8], intestinal tract [9], fat body [10] or ovaries [11]. Besides trehalose absorption, the absorption of other sugars, such as fructose, glucose, and galactose, has been shown for different insect tissues [12]–[17]. Only few examples of sugar transport proteins from insects have been functionally characterized to date [17]–[23]. Except for SCRT, which was classified as a member of family 49 of solute carriers (SLCs) [17], they all belong to the SLC2 family of glucose and polyol transporters [24]–[27]. In principle, SLC2 proteins are integral membrane proteins exhibiting a predicted twelve transmembrane domain topology. The so-called ‘sugar transport signatures’ that signify substrate binding and catalytic activity are also conserved in the SLC2 family. Usually, the expression of these proteins is tissue-specific and responds to metabolic and hormonal regulation. Each of the transporters possesses different affinities for sugars [28].

Although beetles (Coleoptera) represent one of the most diversified lineages on earth with about 350,000 species described and total numbers probably an order of magnitude higher, SLC2 sugar transporters of beetles have not been in the focus of researchers so far. In particular, Chrysomelidae (leaf beetles) constitute, together with the Cerambycidae (longhorn beetles) and the Curculionoidea (weevils), the largest beetle radiation, namely “Phytophaga”, which represent about 40% of all known species [29]. Among phytophagous beetles are many pests such as the mustard leaf beetle *Phaedon cochleariae* which causes yield losses on cruciferous crops in Europe [30]. This species is adapted to use host plants’ leaves as its food source for the duration of its life [31], [32]. Due to its life in an exposed environment, it has to be protected against both the noxious effect of plant secondary metabolites and the attacks by its predators and parasitoids.

When it comes to producing defensive compounds to repel their omnipresent enemies, insect in general are very innovative [33]. To circumvent auto-intoxicative effects, these natural products frequently are processed in exocrine glands [34]–[36]. *P. cochleariae* is known to produce defensive compounds in such glands, herein further referred to as defensive glands, in the larval as well as in the adult stage [37]–[41]. The juvenile beetles possess nine pairs of these glands on their back and release deterrent secretions upon disturbance [42], [43]. Each of these glands is composed of several secretory cells which are attached to a large reservoir. The anti-predatory effect of the secretions can be attributed to cyclopentanoid monoterpenes (iridoids) which are synthesized within the reservoir by few enzymatic reactions from a pre-toxin which is made in the fat body and from there transferred into the defensive glands [44], [45]. Previously it has been shown that defensive gland cells possess ATP-binding cassette (ABC) transporters which are crucial for the shuttling of pre-toxins into the reservoir [46]. Because ATP is used for the cellular metabolism and transport process into the defensive glands, sugars need to be delivered by transporters to drive ATP production in this tissue. Although defensive secretions from phytophagous insects are key players in trophic interactions found in terrestrial food webs [47], their production pathways are often not fully understood. Sugar transporters may be essential components to fuel energy in insect defensive glands, however, nothing is known about their *in vivo* relevance for deterrent production.

Here we focus on a first catalogue of putative members of the SLC2 family as well as trehalose transporters for a phytophagous leaf beetle species. By means of a *de novo*-assembled transcriptome created from the mRNA of *P. cochleariae*, we have performed comprehensive and statistically supported phylogenetic analyses of the identified sequences together with their orthologs selected from other insects and other Metazoa including the known human glucose transporter (GLUT) isoforms. Our data revealed an enormous expansion of the GLUT6/8 sister group in insects and a clade of sequences unique for beetles. Subsequent next generation sequencing-based expression studies revealed putative SLC2 transcripts highly expressed in the defensive glands of juvenile *P. cochleariae*. Single silencing of selected SLC2-candidates by RNA interference (RNAi) resulted only in two cases in a reduced production of defensive secretions. However, in two other cases silencing did not affect deterrent production suggesting an adaptive backup system which stabilizes the sugar level in the defensive glands. To prove the observed homeostasis, we subsequently sequenced the mRNA of silenced larvae to study the differential expression of putative SLC2 transporters not only on phenotypic but also on a transcriptional level.

## Materials and Methods

### Beetles

*P. cochleariae* (F.) was lab-reared on *Brassica rapa chinensis* in a light/dark cycle of 16 h light and 8 h darkness, 14°C±1°C in light and 12°C±1°C in darkness.

### RNA Isolation, Library Construction and Sequencing

Total RNA was isolated from tissue samples from *P. cochleariae* larvae as described by Bodemann *et al*. [48]. Up to 5 µg of total RNA was then used for library preparation using TruSeq™ RNA Sample Prep Kit according to manufacturer’s description. RNA sequencing (RNA-seq) was done using Illumina next-generation sequencing technique [49] on a HiSeq2000 (Illumina, Inc., San Diego, California USA) in 50 bp single-read mode (two or three samples multiplexed in one lane). Pooled total RNAs from the different tissues (such as defensive glands, gut, fat body, Malpighian tubules, and head) from larval *P. cochleariae* were used for paired-end sequencing. Therefore, the fragmentation step during library preparation of the pooled total RNAs was shortened to four minutes (seven minutes for all the remaining samples) to obtain longer fragments. This library was sequenced using a GAIIx (Illumina, Inc., San Diego, California USA) in 150 bp paired-end mode in one sample per lane. All reads were extracted in FastQ format using CASAVA v1.7 (HiSeq) and v1.8 (GAIIx) (Illumina, Inc., San Diego, California USA) for further analysis.

Subsequently to the RNAi experiments (described below), additional sequencing was carried out. Four samples of glandular tissue (one sample of *gfp* dsRNA-injected larvae and three samples of larvae injected with dsRNA of three highly abundant sugar transporters) – two biological replicates each, have been prepared as mentioned above and sequenced on a HiSeq2500 (Illumina, Inc., San Diego, California USA) in 50 bp single-read mode (multiplexed in one lane). All reads were extracted in FastQ format using bcl2fastq v.1.8.3 (Illumina, Inc., San Diego, California USA). The raw sequence data are listed in Table 1 and are stored in the Sequence Read Archive at NCBI (cDNA library 1: SRA100673; cDNA libraries 2: SRA106118, SRA106122, SRA106161; cDNA libraries 3: SRA108012, SRA108036; cDNA libraries 4: SRA108037, SRA108041; cDNA libraries 5: SRA109958, SRA109964; cDNA libraries 6: SRA109966, SRA109967). The corresponding BioProject for *P. cochleariae* can be accessed at NCBI homepage (BioProject ID: PRJNA210148).

**Table 1:** Overview of the raw sequence data.

cDNA library	Tissues for RNA isolation	Total number of reads	Sequencing mode	Remarks
1	gut, fat body, glands, Malpighian tubules	46,030,279	GAIIx 2×150 bp	–
2	glands (3 replicates)	101,383,127	50 bp, HiSeq2000	–
3	glands (2 replicates)	33,013,829	50 bp, HiSeq2500	dsRNA-*gfp* injected
4	glands (2 replicates)	50,926,062	50 bp, HiSeq2500	dsRNA-*Pcsut1* injected
5	glands (2 replicates)	59,678,392	50 bp, HiSeq2500	dsRNA-*Pcsut2* injected
6	glands (2 replicates)	53,204,574	50 bp, HiSeq2500	dsRNA-*Pcsut6* injected

The table exhibits the RNA derived specimens, number of reads, sequencing technology and sequencing mode.

### Transcriptome *de novo* Assembly

The paired-end reads were assembled using Trinity, a RNA-seq *de novo* assembly software [50], [51] with default parameters, minimal contig length of 300 bp and paired fragment length of 500 bp. Afterwards, the *de novo* assembled transcripts were reassembled using the TGI Clustering tools (TGICL), a software program to cluster large EST datasets [52]. The clustering step is performed by NCBI’s megablast [53] and the resulting clusters are then assembled using CAP3 assembly program [54] with following parameters: minimum overlap length of 100 bp and sequence similarity of 90 percent.

### Pfam Analysis

The *de novo* transcripts were translated into their possible protein sequences (all six reading frames) by applying the transeq script which is part of the EMBOSS package (http://imed.med.ucm.es/EMBOSS/). Thereafter, the script pfam-scan.pl (downloaded from the ftp://ftp.sanger.ac.uk/pub/databases/Pfam/Tools/site) was used with showing overlapping hits within clan member families in addition to default parameters to search the protein sequences against the Pfam-A database which consists of high quality protein families based on profile HMMs and clans. A clan is a collection of Pfam-A entries which are related by similarity of sequence, structure or profile-HMM [55], [56].

### Identification of SLC2 Sequences and Trehalose Transporters

The SLC2 sequences and trehalose transporters were identified by searching the Pfam results for Sugar_tr hits. Sugar (and other) transporters are part of the major facilitator superfamily [25] which is believed to function primarily in the uptake of sugars. All identified sugar transporters were searched *via* BLASTp with an E-value smaller than 1e-3 against the Swiss-Prot protein database (ftp://ftp.ncbi.nlm.nih.gov/blast/db/). Swiss-Prot is a high quality and manually annotated and reviewed, non-redundant protein sequence database [57], [58]. For each sugar transporter the top ten hits were inspected, and sequences homologous to known SLC2 or trehalose transporters were identified. SLC2 and trehalose transporters of full-length transcripts or those having a coding sequence of at least 900 bp of length were chosen for further analysis. All studied sugar transporter transcripts are stored in the GenBank database at NCBI as either mRNA (accession numbers: KF803259–KF803269) or Transcriptome Shotgun Assembly (TSA) sequences. This TSA project has been deposited at DDBJ/EMBL/GenBank under the accession GAPU00000000. The version described in this paper is the first version, GAPU01000000. All those sugar transporters were then observed by applying TMHMM [59] and Memsat2 [60] to predict their 12 transmembrane (TM) domains.

### Calculation of Phylogenetic Trees

Phylogenetic trees were calculated for identified SLC2 encoding sequences (see above). Amino acid sequences in multi-FASTA format were aligned using Probalign version 1.4 [61] with default parameters or MAFFT version 7.023b with following settings: –maxiterate 1000 and –localpair. Thereafter, phylogenetic trees were calculated using MrBayes [62], [63] version 3.2.1 and RAxML [64] version 7.2.8 which use different methods. Namely, MrBayes uses Bayesian inference of phylogeny, and RAxML uses maximum likelihood estimation. Applying MrBayes the following settings were used: number of generations was set to 300,000, samplefreq and printfreq were set to 100, the number of runs was set to 2, and the type to calculate the consensus tree was set to allcompat. Applying RAxML the following parameters were used: the model of substitution was PROTGAMMAJTT (GAMMA model of rate heterogeneity), and 1000 rapid bootstrap inferences were done.

For every calculation of the phylogenetic tree only conserved parts were taken into account. Thus, the N-terminus, the loop between TM6 and TM7 as well as the C-terminus were excluded from calculating the phylogenetic tree.

First of all, the trees were calculated only for the chosen sequences derived from *P. cochleariae* to divide these into groups with respect to their sequence similarities. Second, sequences originating from other insects (*Nilaparvata lugens*, *Solenopsis invicta*, *Acyrthosiphon pisum*, *Polypedilum vanderplanki, Bombyx mori*) that have been functionally characterized were added to these sequences (see Table 2) [15], [18], [19], [22]. Trees were re-calculated to identify sequences of *P. cochleariae* that are homologous to the characterized sequences. Third, the human GLUTs as well as other homologous sugar transporters from other Metazoa were selected. The chosen sequences derived from *P. cochleariae* were added to this selection and phylogenetic trees were calculated again to investigate the organisms’ distribution in the trees. Furthermore, we calculated trees for one specific branch (which separated from the others with bootstrap percentage of 100%). For this, orthologous sequences derived from other beetles namely *Dendroctonus ponderosae* [65] and *Tribolium castaneum* were adjoined.

**Table 2:** Characterized trehalose as well as glucose/fructose transporters.

	Description	NCBI accession	Organism
AaSUT_XP_001664193	sugar transporter	XP_001664193.1	*Aedes aegypti*
AgTRET1_BAF96742	trehalose transporter AgTRET1	BAF96742.1	*Anopheles gambia*
AmTRET1_NP_001107211	trehalose transporter 1	NP_001107211.1	*Apis mellifera*
ApTret1like_XP_001950697	fac trehalose transporter Tret1-like	XP_001950697.1	*Acyrthosiphon pisum*
BmTRET1_NP_001108344	fac trehalose transporter Tret1	NP_001108344.1	*Bombyx mori*
DmTRET1–1A	trehalose transporter 1–1, isoform A	NP_610693.1	*Drosophila melanogaster*
DmTRET1–1B	trehalose transporter 1–1, isoform B	NP_725068.1	*Drosophila melanogaster*
DmTRET1–2	trehalose transporter 1–2, isoform A	NP_610694.1	*Drosophila melanogaster*
NlHT1_ABM01870	fac hexose transporter 1	ABM01870.1	*Nilaparvata lugens*
NlSUT1	sugar transporter 1	BAI83415.1	*Nilaparvata lugens*
NlSUT6	sugar transporter 6	BAI83420.1	*Nilaparvata lugens*
NlSUT8	sugar transporter 8	BAI83422.1	*Nilaparvata lugens*
PvTRET1_A5LGM7	fac trehalose transporter Tret1	A5LGM7.1	*Polypedilum vanderplanki*
SiGLUT8	glucose transporter 8	AAX92638.1	*Solenopsis invicta*

They are listed with their description, accession number and organism. Those transporters were added to *P. cochleariae*’s chosen sequences to calculate phylogenetic trees.

### Gene Expression Profiling and Real-time PCR Validation of Putative Sugar Transporters in the Defensive Glands

Three replicates of the cDNA of *P. cochleariae*’s glands were prepared and sequenced as described above. All short reads of three replicates of glandular tissue were mapped onto the reassembled transcripts using Bowtie, an ultrafast short read aligner [66] with –best and –strata options. Bioconductor is an open source, open development software project to provide tools for the analysis and comprehension of high-throughput genomic data. It is based primarily on the R programming language [67]. The mapping results in bowtie format were loaded into R statistics using the ShortRead package [68] which is part of the Bioconductor package. For estimation of variance-mean dependence in count data, the DESeq package was used which is also part of the Bioconductor package (release 2.11) [69], [70]. After analyzing the transcripts with DESeq, the sequences encoding the sugar transporters were selected and sorted regarding the number of sequence counts, beginning with the most abundant reads present in the glands. Furthermore, we normalized the normalized counts to the standards used for quantitative real-time PCR (*Pcrpl6* and *Pcrps3*) by first calculating the normalization factor [71]. The normalized counts were then divided by this normalization factor to get fold changes comparable to the values resulting from quantitative real-time PCR experiments.

Real-time PCR was employed for relative quantification [71]. RNA was isolated as described above. Up to 5 µg of the RNA was reverse transcribed at 50°C for 60 min using SuperScript III and Oligo(dT)_12–18_ primer (life technologies, Darmstadt, Germany). Two technical replicates were performed from three biological replicates each. Technical replicates with a Cq difference of >1 were repeated. To normalize the PCRs for the amount of cDNA template added to the reactions, *Pcrpl6* and *Pcrps3* were chosen for *P. cochleariae* as reference genes. Primers were designed using primer3PLUS: http://www.bioinformatics.nl/cgi-bin/primer3plus/primer3plus.cgi (see Table S1 for primer sequences). Quantitative real-time PCR data were acquired on the CFX96 Touch Real-Time PCR Detection System (Bio-Rad Laboratories GmbH, Munich, Germany) using SYBR Premix Ex Taq II (Tli RNase H Plus) (Takara Bio Inc., Otsu, Japan). Running conditions: 3′ 94°C, 40 cycles [30″ 94°C; 30″ 60°C], melting curve with 1°C increase 60–95°C. These assays were performed following the MIQE-guidelines [72].

### RNA Interference in *P. cochleariae* Larvae

The open reading frames encoding putative transporters of four highly abundant transcripts (*Pcsut1*, *Pcsut2*, *Pcsut5* and *Pcsut6*) were cloned into T7-promotor site lacking TOPO-plasmids pIBV5/HIS (life technologies, Darmstadt, Germany). Plasmids were sequenced prior to further processing. For double stranded RNA (dsRNA) production, sequences of these targets were analyzed *in silico* to avoid sequenced related off-target effects according to [48]. Unique parts of the sequences were amplified with opposing T7-promotor sequences attached to the 5′-end of each forward and reverse primer. The *gfp* sequence was amplified from pcDNA3.1/CT-GFP-TOPO (life technologies, Darmstadt, Germany) accordingly. The amplicons were subject to *in vitro* transcription assays according to instructions from the Ambion MEGAscript RNAi kit (life technologies, Darmstadt, Germany). The resulting dsRNA was eluted from silica membranes after nuclease digestion three times with 50 µl of injection buffer (3.5 mM Tris-HCl, 1 mM NaCl, 50 nM Na_2_HPO_4_, 20 nM KH_2_PO_4_, 3 mM KCl, 0.3 mM EDTA, pH 7.0). The concentration of dsRNA was calculated with A_260_ = 1 = 45 mg/ml and adjusted to 2 µg/µl. The quality of dsRNA was checked by TBE-agarose-electrophoresis.

*P. cochleariae* second instars with 4 mm body length, 0.5–0.7 mg body weight were injected with 0.4 µg of dsRNA about five days after hatching. Injections were accomplished with ice-chilled larvae using a Nano2010 injector (WPI, Sarasota, FL, USA) driven by a three-axis micromanipulator. The larvae were injected dorso-central between the pro- and mesothorax.

According to [45], [73], we calculated the relative growth rate (RGR) of six biological replicates of each group of five larvae by RGR = [(final weight – weight of neonate larva)/(weight of neonate larva×developmental time [days])]. Each replicate group was weighed each 24 or 48 h±3 h and data were compared statistically.

### GC/MS Analysis of Low-molecular-weight Compounds in Defense Secretions

Larval secretions were collected in glass capillaries (i.d.: 0.28 mm, o.d.: 0.78 mm, length 100 mm; Hirschmann, Eberstadt, Germany). Secretions were weighed in the sealed capillaries on an ultra-microbalance (Mettler-Toledo, Gießen, Germany) three times; the weight of the capillaries was subtracted and the final weight was averaged. Sealed capillaries containing samples were stored at −80°C until needed.

According to [48] secretions of *P. cochleariae* were diluted in 1∶200 (w/v) ethylacetate, supplemented with 100 µg/ml methylbenzoate as internal standard. Of each diluted secretion, 1 µl was subjected to GC/EIMS analysis (ThermoQuest Finnigan Trace GC/MS 2000, Frankenhorst, Germany) equipped with Phenomenex (Aschaffenburg, Germany) ZB–5–W/Guardian–column, 25 m. Substances were separated using helium as a carrier (1.5 ml/min). The column temperatures were set as followed: 50°C (2 min), 10°C min^−1^ to 80°C, 5°C mi^−1^ to 200°C, 30°C min^−1^ to 300°C (1 min). Inlet temperature was 220°C and transfer line was 280°C. Chrysomelidial was identified according to [36]. Peak areas of GC-chromatograms were obtained using the ICIS-algorithm (Xcalibur bundle vers. 2.0.7, Thermo Scientific).

### Analysis of Differentially Expressed Genes in Glandular Tissue of RNAi Silenced *P. cochleariae* Larvae

The short reads (sequenced in 50 bp single-mode) from the glandular tissue (4 samples) of the RNAi-silenced *P. cochleariae* larvae have been mapped onto the studied sugar transporters of *P. cochleariae*’s transcriptome using bowtie [66]. The mappings results for the sugar transporters transcripts were subjected to DESeq statistical analysis by reading them into R statistics software, and transcript counts were normalized to the effective library size. Afterwards, the negative binomial testing was carried out to identify differentially expressed transcripts. All those sugar transporters were stringently determined as differentially expressed when having an adjusted p-value smaller than 0.1. Additionally, the normalized counts were stabilized according their variance as outlined in the DESeq package tutorial and heatmaps were generated [70].

## Results

### Identification of Sequences Encoding Putative Members of the SLC2 Family and Trehalose Transporters in the Transcript Catalogue of *Phaedon Cochleariae*

In order to predict sugar transporters in the larvae of *P. cochleariae* with special emphasis on the defensive glands, we performed a comprehensive analysis of transcriptomic data. For this purpose, cDNA libraries prepared from different tissues of juvenile *P. cochleariae* have been sequenced by using the Illumina technique. In addition to the sequencing of cDNA derived from a tissue pool in 150 bp paired-end mode, three biological replicates of the RNA of *P. cochleariae*’s defensive glands were extracted, processed and each sequenced in 50 bp single-read modes. The raw sequence data (in the following called reads) are listed in Table 1. The sequencing of the tissue pool resulted in 46,030,279 read pairs. The *de novo* assembly of those reads by using the Trinity software [50] resulted in 107,323 transcripts with an average sequence length of 796 bp. Reassembly by applying the TGI Clustering tool [52], [74] reduced the number of transcripts to 68,667 with an average length of 929 bp (Table 3).

**Table 3:** Number of assembled transcripts and average length after assembly and reassembly showing the usefulness of reassembling.

	Number oftranscripts	Sum_length	25th_pc	75th_pc	Ave_length
After Trinity assembly	107323	85,475,541 bp	379 bp	841 bp	796 bp
After reassembly with TGICL	68667	63,815,627 bp	383 bp	1037 bp	929 bp

These 68,667 transcripts were then translated into possible protein sequences. The sequences encoding putative sugar transporters were identified by searching the Protein family database (Pfam) [55]. All sequences possessing a Sugar_tr domain (Sugar (and other) transporter family, PF00083) were selected. As a result, a total of 207 sugar transporters could be identified. Those hits were searched via BLASTp with an E-value threshold of 1e-3 against the Swiss-Prot protein database [58]. 68 predicted proteins were annotated as either SLC2 or trehalose transporters (Table S2, Figure S1). These sequences were given temporary designations as numbered series in the form of *Pc*SUTxx (Table S3). According to previous studies, transcripts with a minimum coding sequence length of 900 bp have been chosen for further phylogenetic analyses [20]. The prediction of putative transmembrane (TM) α-helices for those sequences by applying TMHMM [59] as well as Memsat2 [60] revealed that most of our sequences possess 12 TM regions (Table S3, columns 7 and 8). In total, Memsat2 was able to predict those 12 TM regions for 40 of all chosen 68 sequences, whereas TMHMM predicted these for just 33 of all 68 sequences.

### Phylogenetic Analyses of Putative Sugar Transporter Sequences

**Figure 1:**
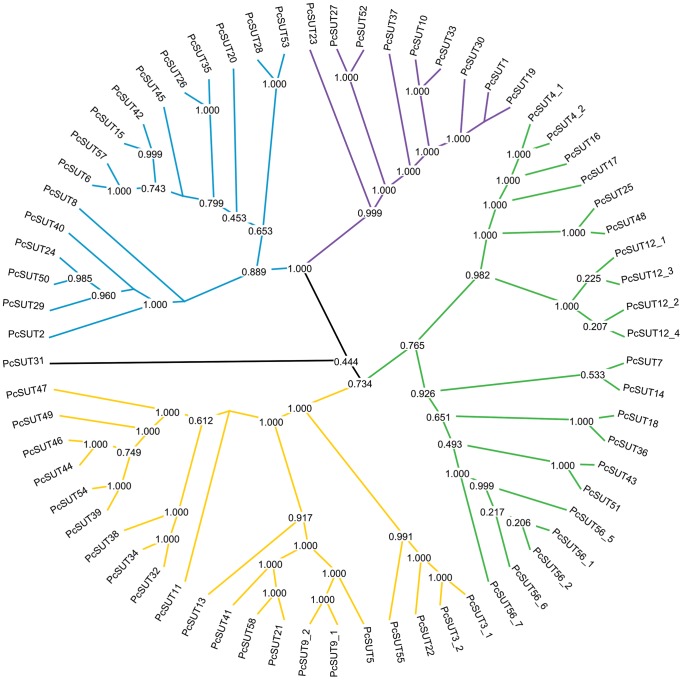
Phylogenetic tree of the 68 chosen sugar transporters derived from *P. cochleariae*. This circular phylogram shows the main 4 groups of chosen sugar transporters. Tree was calculated using MrBayes.

The phylogenetic relationships of our selected 68 transporter sequences were analyzed by applying Probalign (as well as MAFFT [75] for the more complex multiple sequence alignments) for calculating the multiple alignments followed by calculating the phylogenetic trees. Two different methods namely MrBayes and RAxML have been applied. In our case, we could show that both programs resulted in a division of the predicted transporters into at least 4 groups (Figure 1). Especially the purple branch is well supported by a bootstrap value of 100% (Figure S2).

**Figure 2:**
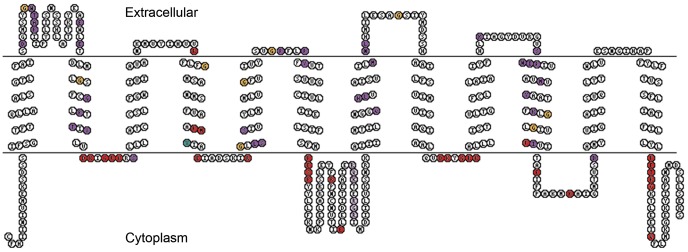
Schematic model for the structure of the putative SLC2 transporters derived from *P. cochleariae* by means of *Pc*SUT1. All 4 groups show the known and conserved facilitated sugar transporter motifs, such as DRxGRR/K in the second loop, PESPR/K in the sixth loop, E/DRxGRR/K in loop 8, and PETK/RGK/R in the carboxy terminal [2], [15], [18], [76], [86]. Furthermore, there are conserved amino acids, such as E and R in loop 4 and 10 (red). Those are needed for the glucose transport activity. Conserved tyrosines (turquois), such as the PMY motif mentioned by Chen *et al*. [15], can be found in our sequences in TMD 4. Additionally, conserved glycines (yellowish) in TMD 1, 2, 4, 5, 7, 8, and 10 as well as in loop 2 and 7 are present, characteristic for the mammalian glucose transporter family. The purple branch exhibits a GWTAP motif in loop 1, a PFYV motif in loop 5, and a VILMNLH motif in TMD 10 (purple colored amino acids).

In general, all 4 groups possess the conserved facilitated sugar transporter motifs important for transport activity and ligand binding, namely DRxGRR/K in the second loop, PESPR/K in the sixth loop, E/DRxGRR/K in loop 8, and PETK/RGK/R in the carboxy terminus (Figure 2, red colored amino acids) [2], [15], [18], [22], [76]. Additionally, conserved tryptophanes in TM domain (TMD) 4, 10 and 11, and loop 10 [77], [78], and conserved tyrosines (turquois colored amino acids in TMD 4), such as the PMY [76], can be found. Furthermore, conserved glycines (yellow colored amino acids) in TMD 1, 2, 4, 5, 7, 8, and 10 as well as in loop 2 and 7 are present, characteristic for the mammalian glucose transporter family [76].

Besides the general conserved amino acid residues, we observed branch specific differences particularly for the purple branch (Figure 2, purple colored amino acids). The conserved motif QQLSG [2] which is present in all branches but substituted with QHXXG in the purple branch is important for the putative substrate binding. In addition, this purple branch exhibits several conserved motifs not present in other branches and not reported from any other transporter of the SLC2 family until now, such as a GWTAP motif in loop 1 (instead of GWTSP), a PFLPFY motif in loop 5, a VILMNLH motif in TMD 7, and a SWIP motif followed by a conserved methionine and tyrosine in TMD 10 (Figure S3, multiple sequence alignment).

**Figure 3:**
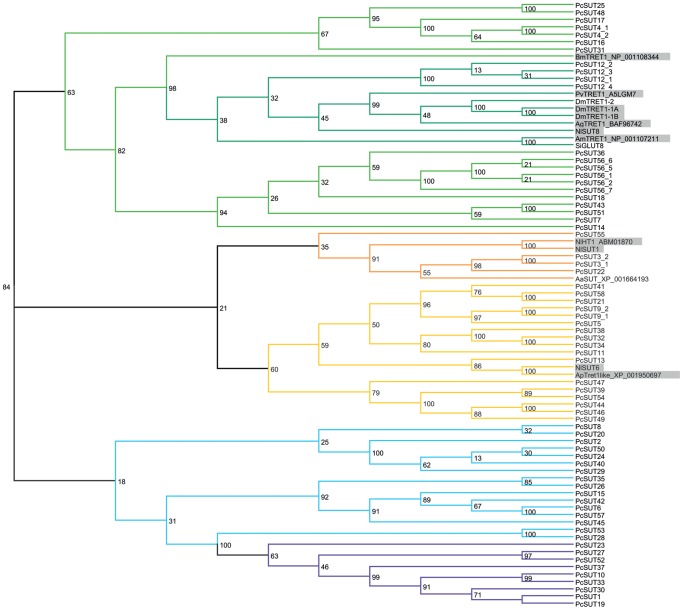
Phylogenetic tree of 68 chosen SLC2 transporters derived from *P. cochleariae* and chosen sugar transporters that have been functionally annotated in various insects (see **Table 2**). This tree was calculated by applying RAxML. The functionally characterized glucose/fructose transporters as well as trehalose transporters from insects are shaded in grey.

By comparing the sequences of chosen sugar transporters to sugar transporters of other insect species that have been functionally characterized [15], [18], [19], [22], we could show that these sequences fall exclusively into the green and yellow branch and not into the purple or blue branch (Figure 3, Figure S4). The functionally proven trehalose transporters such as TRET1 from *Polypedilum vanderplanki*, *Anopheles gambiae*, *Apis mellifera*, *Drosophila melanogaster* and *Bombyx mori* build up a subbranch within the green branch together with *Pc*SUT12_1–4 (with bootstrap percentage of 98%). The GLUT8 of *Solenopsis invicta* also belongs to this group. Therefore, we suggest (in agreement with Kanamori *et al.* [18]), that this fire ant GLUT8 is probably a TRET1 ortholog. The functionally characterized glucose and glucose/fructose transporters from *Nilaparvata lugens* and *Acyrthosiphon pisum* cluster in the yellow branch. However, functional analysis of *P. cochleariae*’s transcripts is required to confirm substrate spectra of the transporters clustering into different branches.

**Figure 4:**
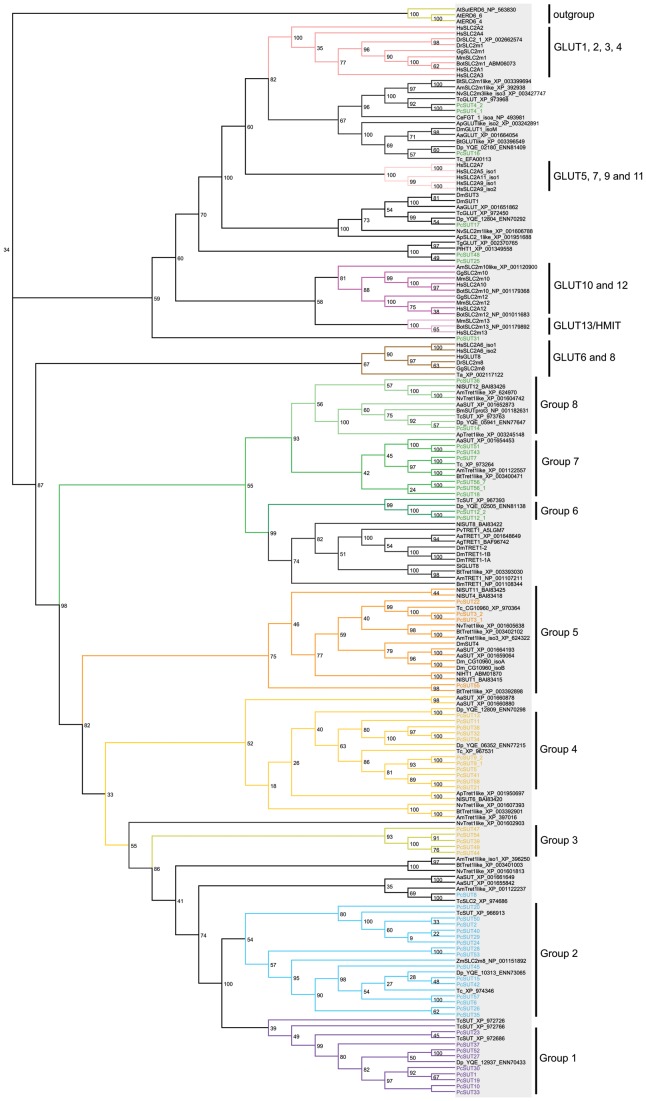
Phylogenetic tree of the *P. cochleariae* sequences and homologous sequences derived from the tree of life calculated using RAxML. Highlighted sequences regard to *P. cochleariae* and most similar sequences. Especially the green branch has to be subdivided into various subbranches, presenting all homologous sequences belonging to Metazoa. The tree significantly shows that the sugars (glucose) and trehalose transporters build up a huge tree in insects. Figure S5 shows the phylogeny of the selected organisms from the tree of life.

To study phylogeny of sugar transporters in a larger context, we selected the *P. cochleariae* sequences and its homologs from selected Metazoa from various branches of the tree of life (Figure S5), including the human SLC2 isoforms (GLUT1–12, H^+^-*myo*-inositol transporter (HMIT)), for cladistic analyses by MrBayes and RAxML. Generally, Figure 4 displays that the sugar (glucose) and trehalose transporters build up a huge branch in insects, and the mammalian sugar porters form a separate branch. In accordance with the published phylogenetic analyses by Wilson-O’Brien *et al*. [79], the mammalian GLUTs isoforms segregate into five distinct classes, also showing that the mammalian GLUTs are separate from their insect orthologs. Some insect sequences also cluster into the mammalian clades, e.g. the class I clade (GLUT1, 2, 3, 4) also contains insect sequences branching at the base with strong support (with bootstrap percentage of 100%). *Pc*SUT31 clusters together with mammalian HMIT. The class II (GLUT5, 7, 9, 11) and the GLUT10/12 clade contain only vertebrate sequences. According to Wilson-O’Brien *et al.* [79], class II genes were most likely to arise after the divergence of this phylum, whereas the GLUT10/12 clade might has been lost in invertebrates secondarily. The majority of the tested insect sequences form a huge sister group of the GLUT6/8 class with strong support (100% bootstrap), suggesting an expansion of this class in insects. On inspection of the insect sequences, especially the green branch is remarkably large and can be subdivided into various subbranches. In addition to the five mammalian SCL2 classes, we suggest eight more classes in insects (each separated from the others with bootstrap percentages of at least 80%).

**Figure 5:**
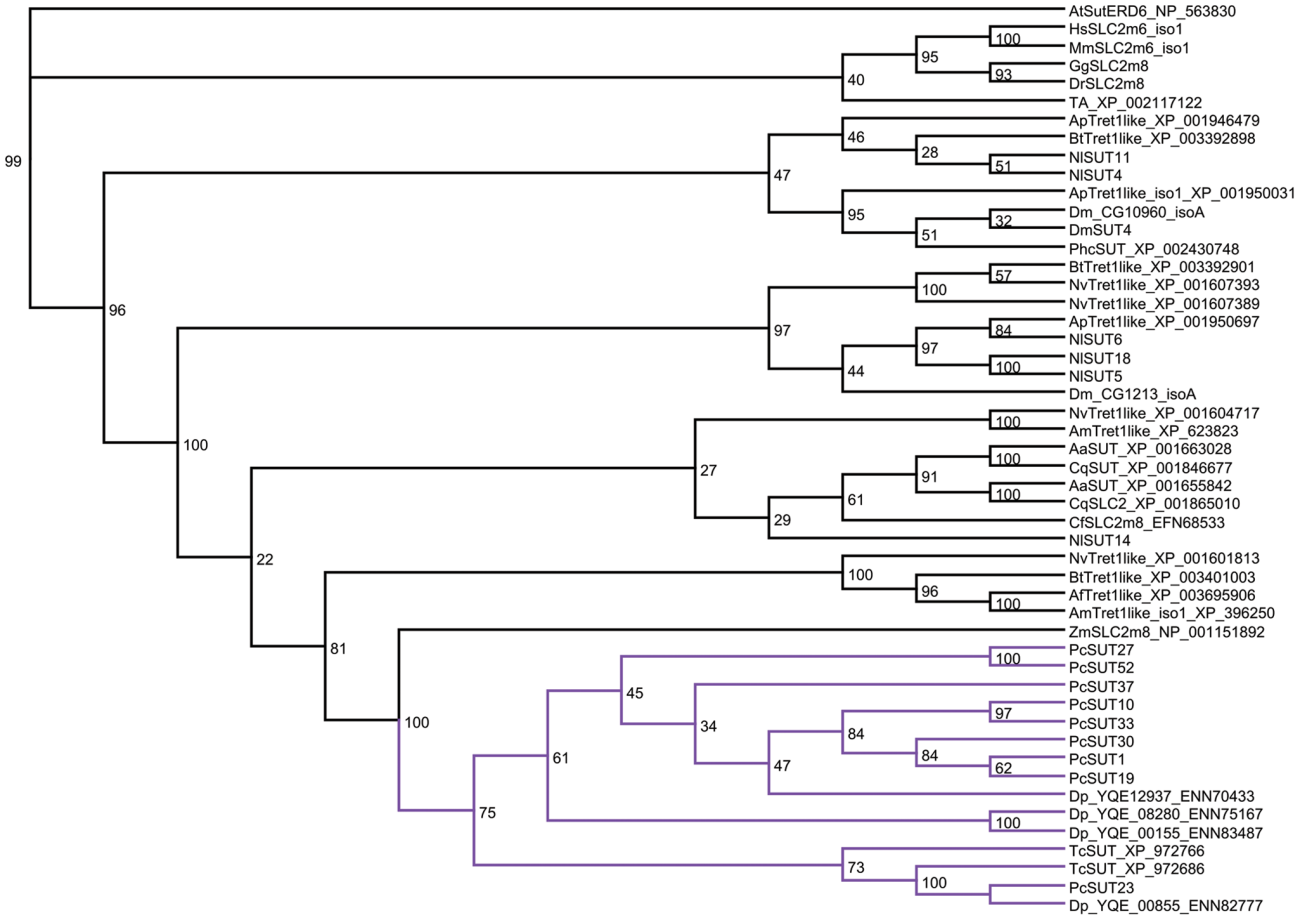
The phylogenetic tree of the nine sequences derived from *P. cochleariae* belonging to the purple branch (see **Figure 3** and **4**, Figure S4) and homologous sequences derived from the whole tree of life, especially from *Dendroctonus ponderosae* (Dc) as well as from *Tribolium castaneum* (Tc), was calculated using RaxML. Indicated in purple, it can be seen that the beetles’ sequences build up a separate branch.

The bootstrap values of the tree of life support the notion that the purple branch may be restricted to the insect order Coleoptera. For deeper analysis of this branch, we have calculated trees with the sequences of this purple branch and its homologs/orthologs in insects. The MrBayes tree as well as the RAxML tree (Figure 5 and Figure S6) shows that only sequences derived from beetles such as *Dendroctonus ponderosae*, *Tribolium castaneum,* and *P. cochleariae* belong to this purple branch. Whereas, looking at Figure 3 and 4, all other sequences derived from *P. cochleariae* show homologies to Diptera, Apocrita and other insect orders. According to our cladistic analyses, the purple branch of the phylogenetic trees seems to be the most interesting. All changed and additionally conserved motifs together with the motif QH lead to the hypothesis that the sequences belonging to the purple branch may provide a new class of sugar transporters in insects, especially in the order Coleoptera, not before described. However, their biological function remains to be elucidated.

### Expression Profiles of SLC2 and Trehalose Transporter Transcripts in the Defensive Glands of Immature *P. cochleariae*

The Illumina short reads derived from the glandular tissue sequenced using HiSeq2000 in 50 bp single-read mode (two or three samples multiplexed in one lane) resulted in 34,918,295 reads with length of 50 bp as well as 36,598,828 and 29,866,004 short reads, respectively. Those short reads have been mapped onto our transcriptome. 82.62% to 84.90% of all short reads could be mapped, whereas 14.28% to 16.31% of the reads failed to align (Table S4).

**Figure 6:**
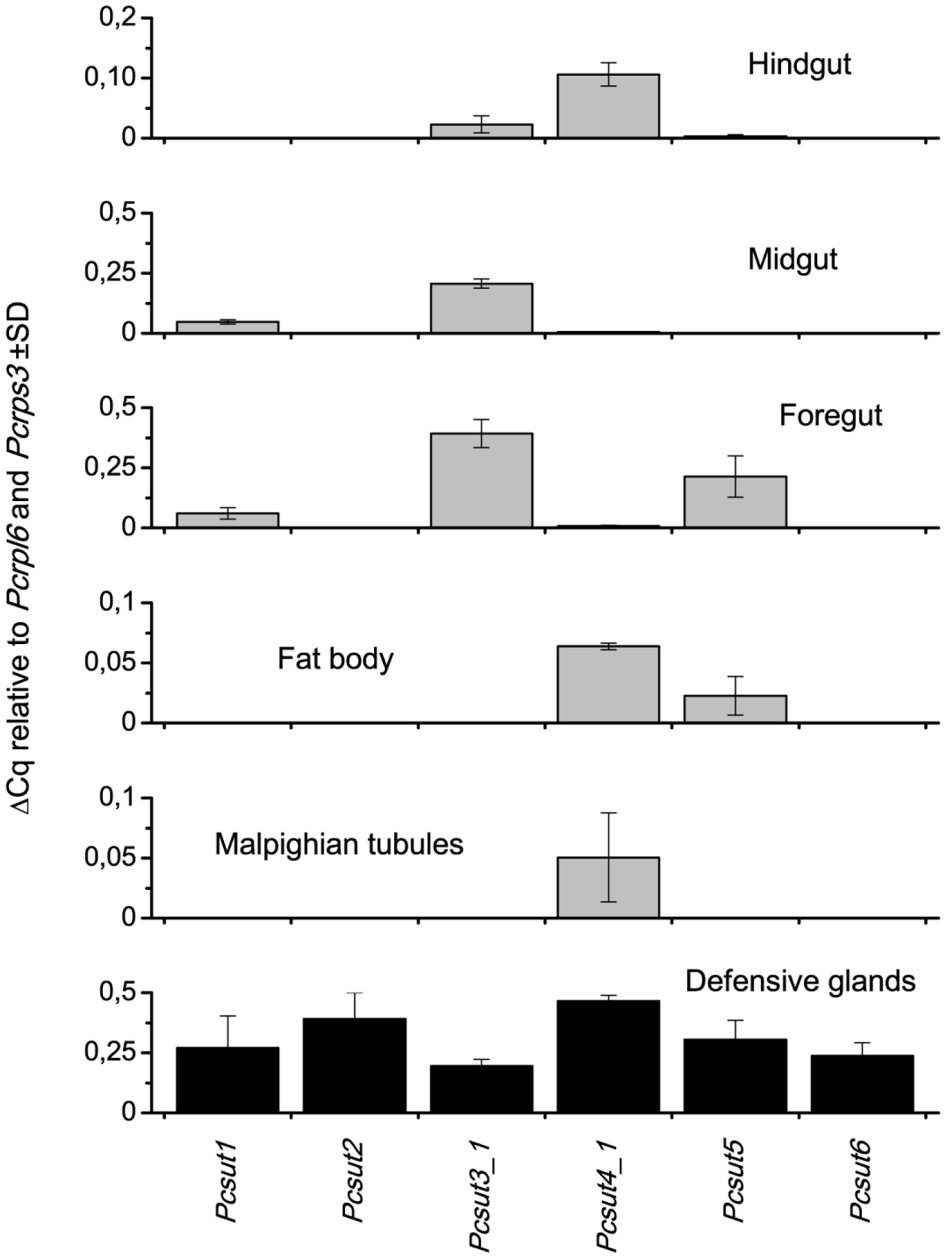
Distribution of mRNA levels of putative SLC2 transporters in various tissues of juvenile *P. cochleariae* by using quantitative real-time PCR.

The mapping results were taken as input for R statistics. DESeq, belonging to the Bioconductor package, was applied to analyze the mapping results. The counts of transcripts are listed in Table S3 (columns 3 to 5). All observed putative sugar transporters of SCL2 and trehalose transporters could be identified in the glandular tissue, although most of them at a very low level. Interestingly, the six most abundant transcripts (inclusive isoforms) with more than 3,000 normalized counts are spread among all four major phylogenetic subtrees as highlighted in Figure S7. Therefore, no specific branch is particularly overrepresented in the defensive glands. As previously demonstrated in literature [80], evaluation of the RNA-seq data (standardized values shown in Table S5) with quantitative real-time PCR data shows also in our experiments the comparability of the two methods (Figure S8). Subsequently, the six transcripts have been analyzed regarding the distribution of their transcript levels in different larval tissues. Two sugar transporters namely *Pc*SUT2 and *Pc*SUT6, clustering into the blue branch, are exclusively expressed in defensive glands (Figure 6). The other four tested candidates were found to be also expressed in at least one more tissue.

### Silencing of Putative Sugar Transporters in Immature *P. cochleariae* by RNAi

**Figure 7:**
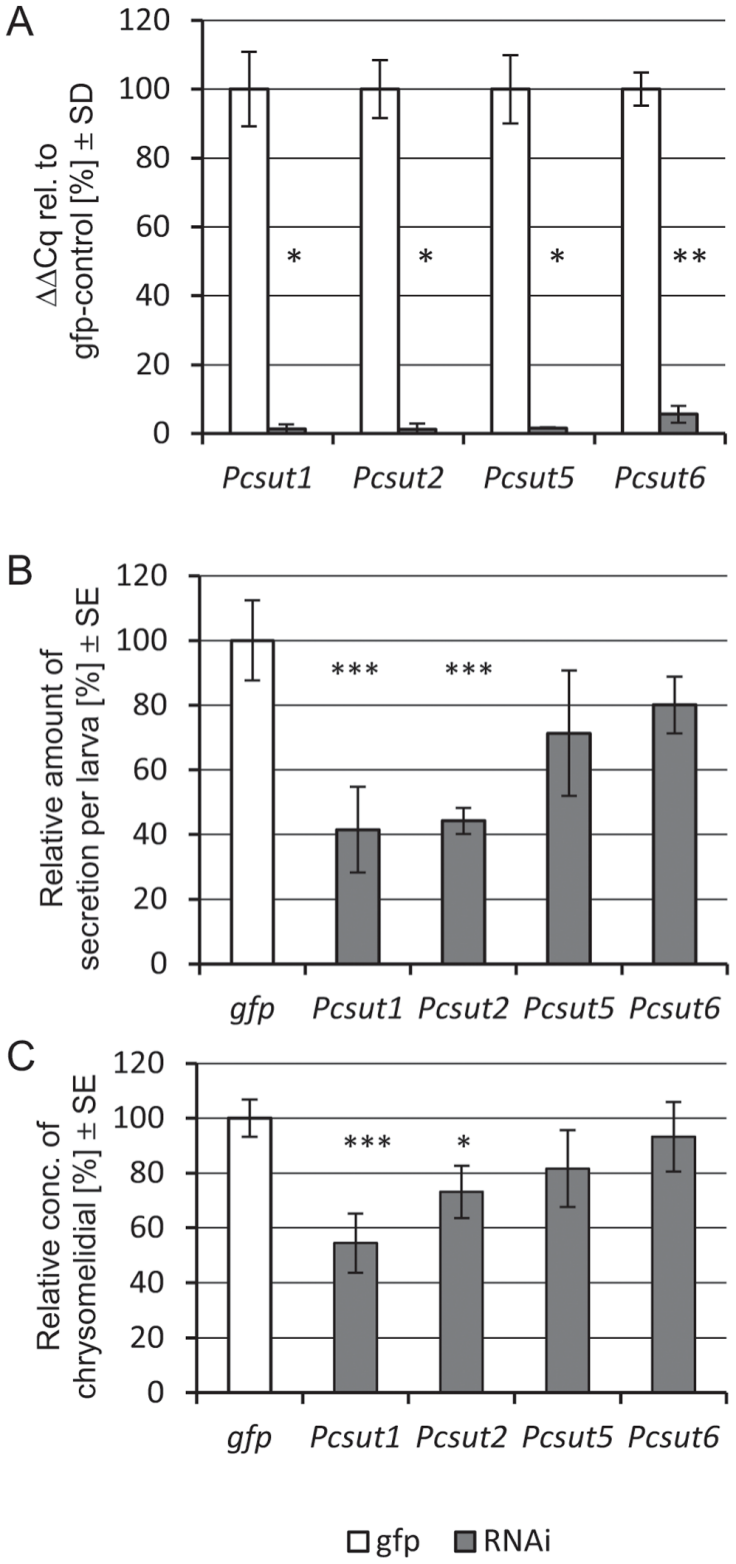
RNAi effects on transcript levels, amounts of defense secretions and chrysomelidial concentrations 10 days post RNAi induction in juvenile *P. cochleariae*. A, Relative expression of chosen transporters in glandular tissue, normalized internally to *Pcrpl6* and *Pcrps3* and externally to *gfp*-control, n = 5. B, Amounts of secretions produced by individual larvae were weighted and normalized to the control treatments. n = 5. C, Secretions samples of RNAi induced larvae were analyzed using GC/MS; Amounts of chrysomelidial were normalized to internal standard (methylbenzoate), values were calculated against control. n = 5. Asterisks indicate level of significance (T-test, 2-tailed; p-value< = 0.05 = *, < = 0.01 = **, < = 0.001 = ***).

RNA interference (RNAi) was carried out, besides for *Pcsut2* and *Pcsut6*, also for *Pcsut1* (highly expressed in defensive glands and gut) and *Pcsut5* (highly expressed in defensive glands, fat body and gut) to get deeper insights into their biological function for the development of *P. cochleariae* larvae and the production of defensive secretions *in vivo*. Early second instars were injected with either dsRNA identical to a unique part of one of these transporters sequences or with dsRNA of *gfp.* As confirmed by quantitative real-time PCR, transcription of all targets was successfully silenced in the defensive glands 10 days after injection (Figure 7A). By monitoring the development of treated larvae, significant weight reduction was observed neither for the larvae nor the pupae by transporter silencing (Figure S9). To screen for the function of the transporters for the synthesis of secretions, GC/MS analysis was carried out for quantification of the amount of chrysomelidial relatively to the *gfp*-controls. Here, we could observe decreases in the amount of chrysomelidial by silencing *Pcsut1* (p = 0.008) and *Pcsut2* (p = 0.001) and also in the amount of secretions produced by targeting *Pcsut1* (p = 0.007) and *Pcsut2* (p = 0.03) (Figure 7B and 7C). Knocking down of neither *Pcsut5* nor *Pcsut6* resulted in alterations of the phenotype. We assume that *Pc*SUT1 and *Pc*SUT2 seem to be important for the production of defensive secretions. Their substrate selectivity, however, needs to be further studied *in vitro*.

In contrast to the silencing effect of the ABC transporter in the defensive glands which caused a total loss of defensive secretions [46], RNAi targeting SLC2 transporters could not shut down the production of defensive exudates completely. This may be due to the turn-over rate of the integral-membrane proteins which may diminish the silencing effect or other transporters may be expressed to take over the function to achieve homeostasis in the tissue. To test the latter hypothesis, we have gathered RNA-seq data from larvae silenced in *Pcsut2* or *Pcsut6* (as examples for SLC2 members exclusively expressed in glands) or *Pcsut1* (as example highly expressed in defensive glands and gut) and analyzed differential expression of SLC2 transporters in comparison to *gfp*-treated larvae.

### Analysis of Differentially Expressed Genes in the Glandular Tissue of RNAi-silenced *P. cochleariae* Larvae

Ten days after *Pcsut1, Pcsut2* and *Pcsut6*-silencing and ds*gfp*-injection, glandular tissues were dissected and two biological replicates for each treatment were sequenced. The normalized counts of all sugar transporters of all samples are listed in Table S6. The log_2_ fold-changes of the silenced transporters (ds*gfp*-injected samples as control) and adjusted p-values were determined using the DESeq package (see Table 4). In Figure S10 all sugar transporters exhibiting significantly different transcription levels are colored red (MA plot showing log_2_ fold-changes *vs.* mean values). In all samples (prepared in RNA-seq and quantitative real-time PCR experiments), we observed varying transcript levels of SCL2 transporters owing to the individual biological variance and diversity despite similar developmental stage or living conditions.

**Table 4:** Differential expression analysis using DESeq package.

Differentially expressed transcripts with padj<0.1 after dsRNA injection of *Pcsut1*:						
Seq_Id	baseMeanA	baseMeanB	foldChange	log_2_FoldChange	pval	padj
PcSUT1	4804.953616	240.8731589	0.050130174	−4.31817696	1.26E-16	7.31E-15
**Differentially expressed transcripts with padj<0.1 after dsRNA injection of ** ***Pcsut*** **2:**						
**Seq_Id**	**baseMeanA**	**baseMeanB**	**foldChange**	**log_2_FoldChange**	**pval**	**padj**
PcSUT2	8428.420254	2031.419654	0.241020214	−2.052773946	4.15E-08	2.49E-06
PcSUT25	22.79115587	394.3587471	17.30314817	4.112962644	0.003061159	0.091834771
**Differentially expressed transcripts with padj<0.1 after dsRNA injection of ** ***Pcsut*** **6:**						
**Seq_Id**	**baseMeanA**	**baseMeanB**	**foldChange**	**log_2_FoldChange**	**pval**	**Padj**
PcSUT6	3380.778995	95.65776606	0.028294593	−5.143329799	4.20E-14	2.56E-12
PcSUT25	22.79115587	995.628527	43.68486322	5.449061568	0.000272434	0.008309243

baseMeanA: mean of normalized counts value of ds*gfp*-injected samples. baseMeanB: mean of normalized counts values of dsRNA-*gfp*-injected, dsRNA-*Pcsut1*-injected, dsRNA-*Pcsut2*-injected, dsRNA-*Pcsut6*-injected samples. Fold-change: baseMeanA compared to baseMeanB. Log_2_fold-change: logarithm (to base 2) of fold-change values. Pval: p-value for the statistical significance of this change. Padj: p-value adjusted for multiple testing with Benjamini-Hochberg procedure which controls false discovery rate.

**Figure 8:**
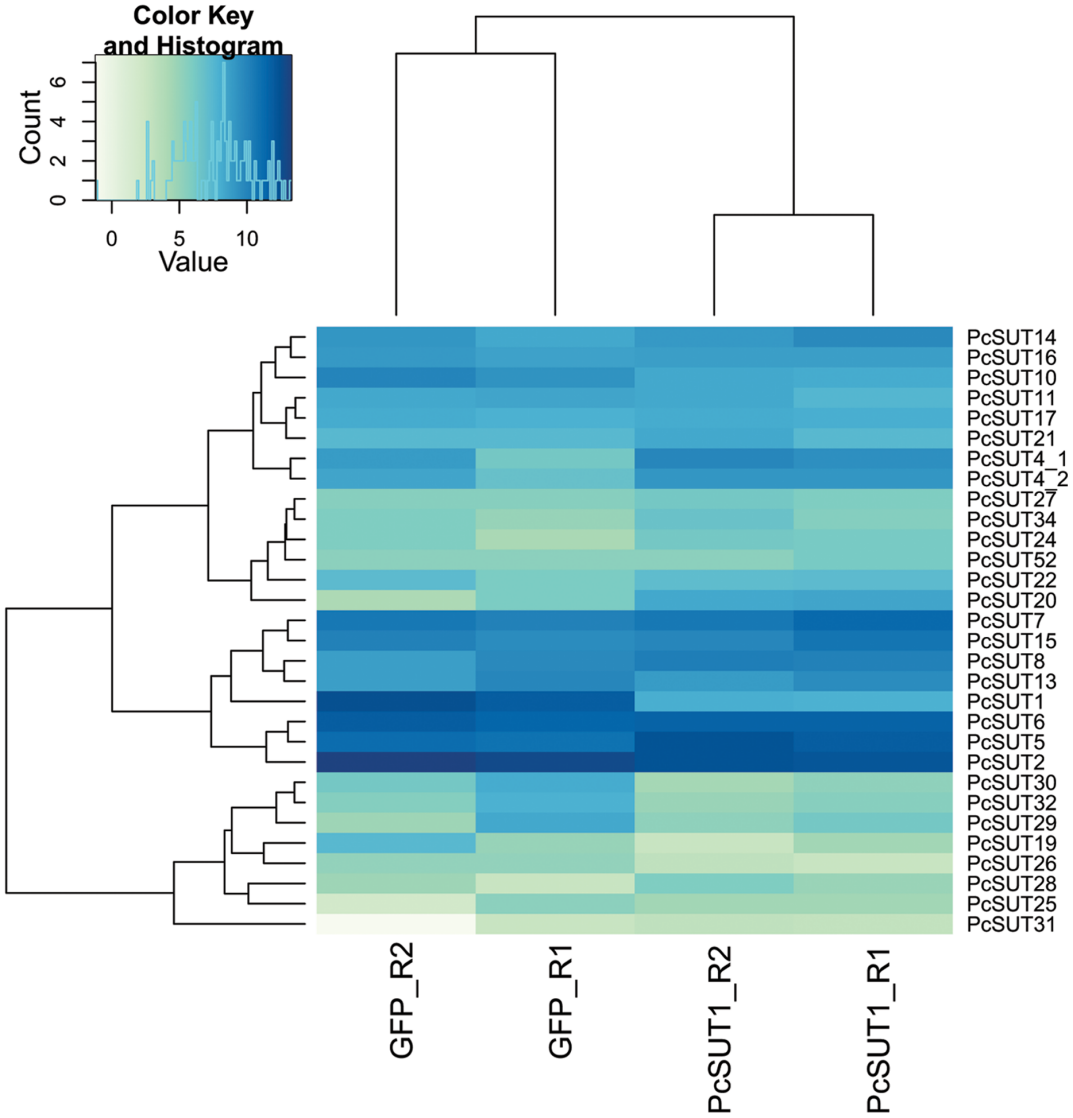
Heatmap of the variance stabilization transformed data (vsd) of ds*Pcsut1*-injected *vs*. ds*gfp*-injected samples. Samples derived from glandular tissue. For this, the transcript counts of the sugar transporters of each sample after dsRNA-injection have been normalized to the effective library size and the variance over all samples has been stabilized by applying the DESeq package. For each heatmap, the 30 most abundant sugar transporter transcripts are shown. Ds*gfp*-injected samples are the same in each heatmap.

*Pcsut1* knocking-down led to a significant decrease of its own transcript level (Table S6: adjusted p-value (padj) = 7.31E-15). Figure 8 exhibits a heatmap of the 30 most abundant sugar transporters. Besides *Pcsut1*, three more sugar transporters were co-silenced, namely *Pcsut10*, *Pcsut30* and *Pcsut32*. Those were not determined as differentially expressed, but have a log_2_ fold-change smaller −2 (Figure S10A, lower right quadrant). In contrast, *Pcsut4*, *Pcsut5*, *Pcsut20*, and *Pcsut22* were up-regulated (log_2_ fold-change of 1) suggesting counter-regulation to the silencing effect to ensure sugar homeostasis in the defensive glands. This up-regulation, however, did not fully compensate the silencing effect of *Pcsut1* indicated by the decrease in the production of defensive secretions (as shown before).

**Figure 9:**
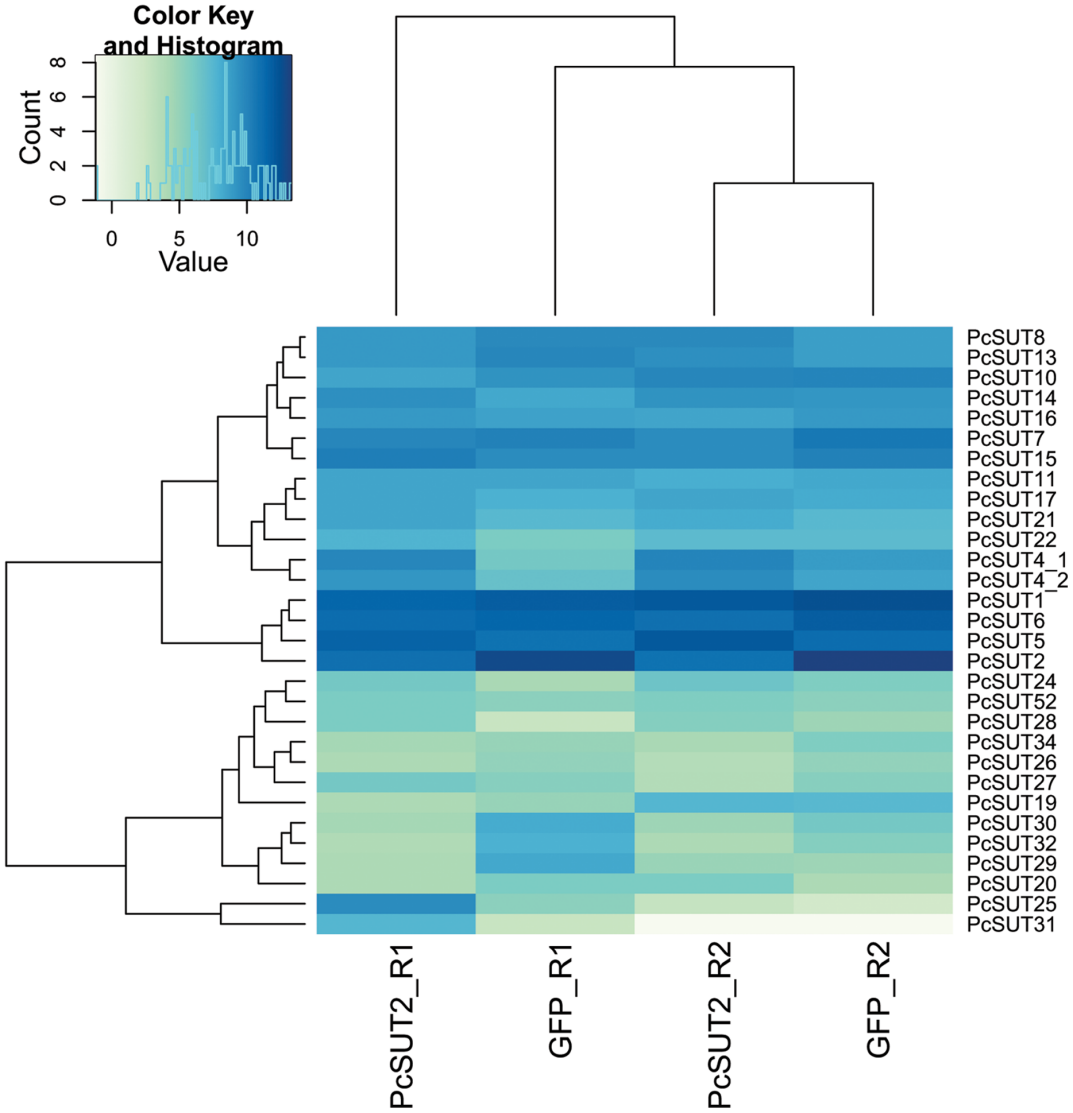
Heatmap of the variance stabilization transformed data (vsd) of ds*Pcsut2*-injected *versus* ds*gfp*-injected samples. Samples derived from glandular tissue. For further explanation see Figure 8.

The samples prepared after dsRNA-injection of *Pcsut2* showed, on the one hand, a significant down-regulation of *Pcsut2* itself (padj = 2.49E-06), but also a decrease of *Pcsut6*, *Pcsut26*, *Pcsut30*, *Pcsut32* and *Pcsut34* (log_2_ fold-change of −1, Figure S10B lower right section). On the other hand, *Pcsut4*, *Pcsut5*, *Pcsut14*, *Pcsut17*, *Pcsut21*, *Pcsut24* and *Pcsut28* were up-regulated (Figure 9). Additionally, the transcript level of *Pcsut25* was determined as significantly higher in the silenced samples than in the ds*gfp*-injected samples (padj = 0.092) (Table 4). But also here, the counter-regulated transporters did not compensate the silencing effect completely.

**Figure 10:**
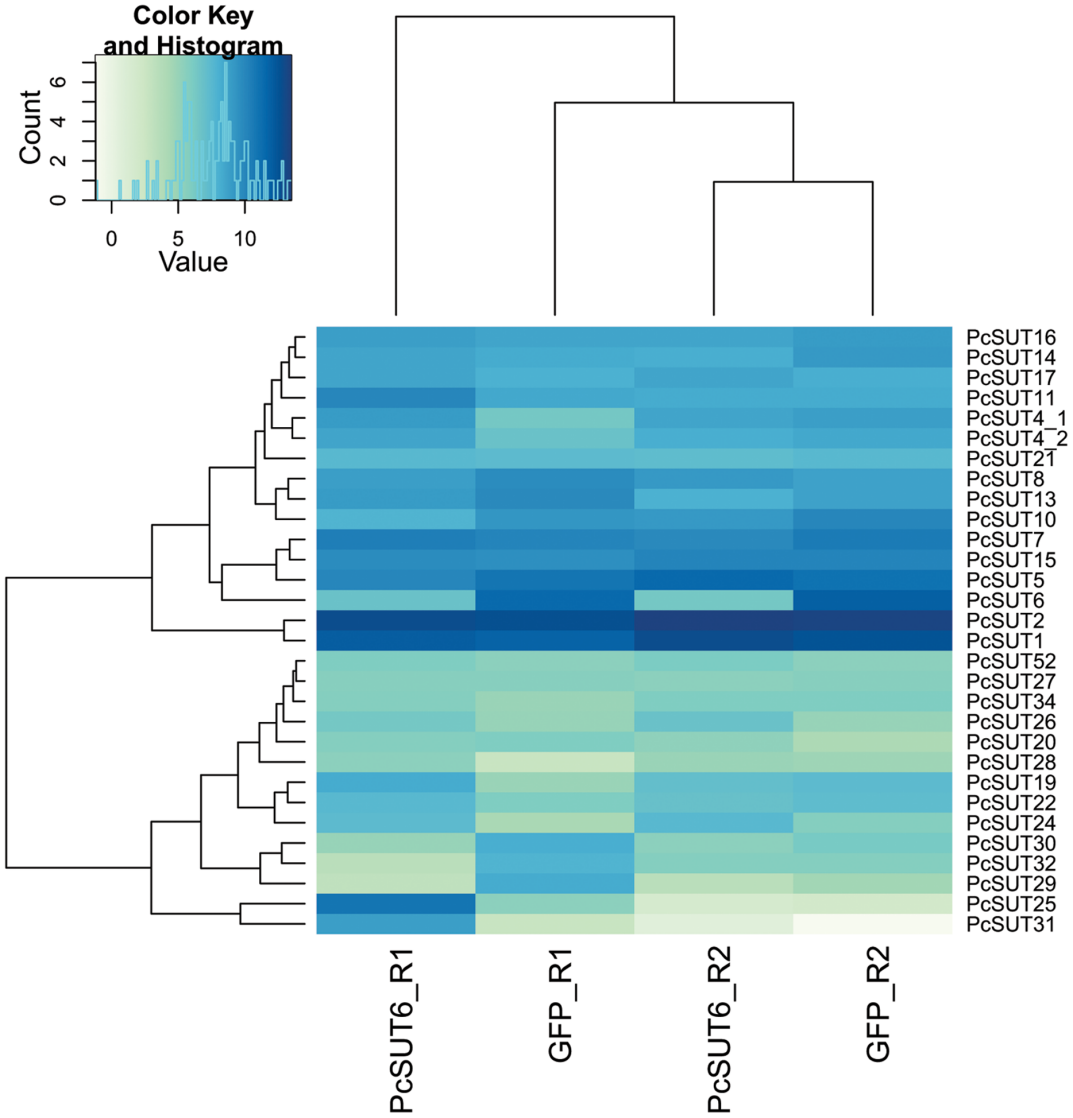
Heatmap of the variance stabilization transformed data (vsd) of ds*Pcsut6*-injected *vs*. ds*gfp*-injected samples. Samples derived from glandular tissue. For further explanation see Figure 8.

In the samples with *Pcsut6* silenced *via* dsRNA-injection, *Pcsut6* was drastically reduced (padj = 2.56E-12). Furthermore, *Pcsut13*, *Pcsut29*, *Pcsut30* and *Pcsut32* were also down-regulated (log_2_ fold-change of −1, Figure S10C). To establish the sugar homeostasis in this sample, *Pcsut1*, *Pcsut17*, *Pcsut24* and *Pcsut26* were transcribed at a higher level (log_2_ fold-change of 1) compared to the ds*gfp*-injected samples. Especially *Pcsut25* was significantly higher expressed (padj = 0.008) (Figure 10, Table 4).

## Discussion

Sugars play an important role in all species’ metabolism. Transporters of the SLC2 family are key elements involved in the adaptive response to sugar demand that have important physiological implications to cell survival and growth. They are expressed in a tissue-specific manner with different affinity, specificity and capacity for substrate transport [81]. Recent phylogenetic analyses of genomic data available from sequenced insects suggest a remarkable expansion of the SLC2 family in insects compared to mammals [18], [20], [23], [26], [79]. Beetles, however, have not yet been addressed. Here we present the first comprehensive phylogenetic analysis of members of the SLC2 sugar transporter family identified in a leaf beetle species.

### Phylogenetic Analyses

We created a transcript catalogue of juveniles of *P. cochleariae* in which we annotated 68 sequences as SLC2 transporters. The phylogenetic analyses of putative sugar transporters were performed with MrBayes and RAxML. MrBayes, on the one hand, is a program for Bayesian inference and model choice across a large space of phylogenetic and evolutionary models. On the other hand, RAxML is a maximum likelihood phylogeny estimation. In our case, we were able to show that both programs result in a division of the predicted transporters into 4 main groups. However, there are some differences in the results of the calculations with the two methods. For example, *Pc*SUT8 belongs to the green branch of the phylogenetic tree showing *P. cochleariae*’s sequences as well as the functionally characterized sequences of the other insects calculated by MrBayes; RAxML sorted it into the blue branch (Figure 3, Figure S4). Comparing these trees with the trees showing only sequences of *P. cochleariae* as well as with the trees which also include the sugar transporter sequences of mammals and other organisms, it can be seen that *Pc*SUT8 always belongs to the blue branch. To conclude, we propose that RAxML results in more stable trees when adding or deleting homologous sequences. This fact might be strengthened by Douady *et al.* [82] who concluded that the more conservative use of bootstrap percentages (as used by RAxML) might be less prone to supporting strongly a false phylogenetic hypothesis.

The mammalian GLUT proteins are well-studied, and phylogenetic studies have been carried out. In addition to the five distinct classes of mammalian GLUTs [79], we propose eight more groups of transporters including the trehalose transporters in the Insecta. The tree including mammalian GLUTs as well as homologous sequences of other organisms including insects shows that many transporters belonging to the SLC2 family derived from *P. cochleariae* and other insects constitute a huge subtree separate from the well-studied mammalian GLUTs. We suggest that the orange branch within this insect clade constitutes the fructose/glucose transporters. This suggestion is supported by, on the one hand, *Ap*ST3 from the pea aphid *A. pisum* which acts in the gut as a low-affinity uniporter for fructose and glucose [23], and on the other hand, *Nl*HT1 (*Nl*ST1) and *Nl*ST6 from the brown planthopper *N. lugens* which function as glucose and glucose/fructose transporters, respectively, in the gut [20], [22]. The green branch within the huge insect clade most likely contains trehalose transporters including the transporter TRET1 from the sleeping chironomid *P. vanderplanki* and its orthologs from *A. gambiae*, *B. mori*, *A. mellifera* and *D. melanogaster* [18], [19] and a proton-dependent transporter participating in trehalose reabsorption in Malpighian tubules of *N. lugens* [21]. Kinetic parameters show different affinities for trehalose among the TRET1 orthologs which mirror the trehalose:glucose ratio in the hemolymph of each species [18]. No functional assumptions can be made regarding all other subbranches, especially concerning the purple one which seems the most interesting to the study of beetles.

The large number of SLC2 transporters may result from gene duplications as has been suggested for *A. pisum,* whose genome contains a conspicuous number of genes encoding predicted sugar transporters [23]. While such an idea still needs experimental proof, it may contribute to the reflection on how insects are able to adapt to extreme dietary conditions and to the testing of substrates not yet in the focus for SLC2 members such as plant derived glucosides, for example, present in large amounts in the diet of phytophagous insects.

Interestingly, the mammalian GLUT6/8 form a sister group of the expanded insect clade. In accordance with the literature [76], [79] we see that GLUT6 and 8 are more closely related to sugar transporters present in invertebrate species than to other mammalian GLUTs. Mueckler *et al*. [27] stated that the primary physiological substrate for mammalian GLUT6 and 8 have not been definitely identified. Therefore, knowledge of substrate selectivity of the SLC2 members in the insect clade may also contribute to a deeper understanding of the function of GLUT 6 and 8 in mammals.

### Membrane Protein Topology

Membrane proteins, such as sugar transporters, seem to have a restricted range of folds than their water-soluble counterparts, making them more amenable to structural predictions [83]. α-Helical membrane proteins contain one or more transmembrane helices which consist predominantly of hydrophobic amino acids. In our study of sugar transporters, 12 TM helices are stated [26]. To predict the TM domains of *P. cochleariae*’s sugar transporters, TMHMM as well as Memsat2 were used. For 40 of 68 sequences, Memsat2 was able to predict all 12 TM domains. TMHMM predicted those 12 TM domains for just 33 sequences. But, for three sequences Memsat2 failed to predict at least 10 TM helices and proposed 1 (twice) and 2 (once) TM helices. For those three sequences, TMHMM predicted 10 (once) and 12 (twice) TM helices. This leads us to the conclusion that neither of these prediction methods is perfect, but applying both gives us the required information. Furthermore, Cuthbertson *et al.* [83] suggested that optimal prediction is obtained by the method that best reflects the biological and physical principles governing membrane protein architecture.

### RNAi and Subsequent RNA-seq

After silencing *Pcsut1* and *Pcsut2*, phenotypic analyses revealed a reduction of defensive exudates in the larvae. Silencing of *Pcsut5* and *Pcsut6* did not result in a changed phenotype. By combining the RNAi experiments (silencing *Pcsut1*, *Pcsut2* and *Pcsut6*) with subsequent mRNA isolation and RNA sequencing, we could show the down-regulation as well as up-regulation of sugar transporters in the defensive glands. Silenced and induced sequences belong to separate branches in the phylogenetic trees which also suggest that insect transporters belonging to different clades can have comparable substrate preference. Anyhow, a direct correlation of counter-acting transporters was difficult to identify. All predicted off-targets according to Bodemann *et al.* [48] were excluded in the transporter sequences used for RNAi. Nevertheless, co-silencing effects could not be avoided. These effects could not be predicted most likely because of metabolic co-silencing which was already observed for two hexokinases in *T. castaneum* [84].

Homeostasis may not only be achieved by the induced expression of transporters but, for example, also by the induced trafficking of transport proteins within a cell. This trafficking is known, for example, from the mammalian GLUT4. The protein is hormonally induced to translocate from intra-cellular membranes to the plasma membrane for the absorption of excessive glucose from the blood [85]. In insects, however, an analogous phenomenon is not known and was not addressed in our analyses. In general, sugar homeostasis is not very well understood in insects, and the role of SLC2 members in this process has not been addressed to date. By transporter silencing in the defensive glands of the mustard leaf beetle larvae, we conclude that there is a complex network of SLC2 transporters in which several transporters compensate the function of the silenced ones. We demonstrate clearly the potential of SLC2 transporters to respond adaptively to nutrient demand. This response may have paramount ecological implications for the survival of phytophagous beetles in plant-insect interactions.

## Supporting Information

Figure S1 **Bar plot showing the molecular functions assigned to the** ***Pc*** **SUTs.** First, BLASTx was performed to annotate the studied sugar transporters of *P. cochleariae*. Thereafter, BLAST2GO was applied to those BLAST hits. The assigned molecular functions are displayed.
(EPS)

Figure S2 **Phylogenetic tree of the 68 chosen sugar transporters derived from** ***P. cochleariae*** **.** The phylogenetic tree was calculated with RAxML showing the main 4 groups. The purple subbranch is separated from the other branches with a bootstrap value of 100%.
(EPS)

Figure S3 **Multiple sequence alignment of the 68 chosen sugar transporters derived from** ***P. cochleariae*** **.** The multiple sequence alignment was calculated using Probalign. The purple-branch-specific amino acids are framed.
(TIF)

Figure S4 **Phylogenetic tree of** ***Pc*** **SUTs derived from** ***P. cochleariae*** **and functionally characterized sugar transporters of other insects listed in** **Table 2** **calculated with MrBayes.**
(EPS)

Figure S5 **Phylogenetic tree showing the selected organisms from the tree of life.**
(EPS)

Figure S6 **The phylogenetic tree of the nine sequences derived from** ***P. cochleariae*** **belonging to the purple branch (see** **Figure 3** **and** **4** **, Figure S4) and homologous sequences derived from the whole tree of life, especially from** ***Dendroctonus ponderosae*** **(Dc) as well as from** ***Tribolium castaneum*** **(Tc), was calculated using MrBayes.** Indicated in purple, it can be seen that the beetles’ sequences build up a separate branch.
(EPS)

Figure S7 **Phylogenetic** ***tree*** **of the 68 chosen sugar transporters derived from** ***P. cochleariae*** **.** The six most abundant glandular sugar transporters are circled and not branch-specific, but distributed all over the tree.
(EPS)

Figure S8 **Relative mRNA levels of putative SLC2 transporters in the defensive glands of juvenile** ***P. cochleariae*** **determined by carrying out RNA-seq (A) and quantitative real-time PCR (B) experiments.** The corresponding fold-changes of the RNA-seq samples are listed in Table S5.
(TIF)

Figure S9 **RNAi effects on the development of the larvae from** ***P. cochleariae*** **.** A, The development of larval weight was documented and measured in a 24 or 48 h±3 h period. B, In neither the relative growth rate nor in the weight of freshly emerged pupae significant differences could be observed between ds*gfp-* and ds*Pcsut1-,* ds*Pcsut2-,* ds*Pcsut5-,* ds*Pcsut6*-injected larvae, n = 30.
(TIF)

Figure S10 **MvA-Plot showing normalized mean values** ***versus*** **log_2_fold-changes.** The fold-changes (log-transformed) were computed for the comparison of ds*gfp*-injected and ds*Pcsut1*-injected samples. The transcript levels are significant at 10% FDR (padj< = 0.1, padj: p-value adjusted for multiple testing with the Benjamini-Hochberg procedure which controls false discovery rate (FDR)). The differentially expressed transporters, the ones colored red, are listed in Table 4. (A) MvA-Plot of the comparison of ds*Pcsut1*-injected and ds*gfp*-injected samples. *Pcsut1*’s transcript level was significantly reduced by RNAi (red dot in the lower right quadrant). (B) MvA-Plot of the comparison of ds*Pcsut2*-injected and ds*gfp*-injected samples. *Pcsut2*’s transcript level was significantly reduced by RNAi (red dot in the lower right area). Additionally, the expression of *Pcsut25* was significantly induced (red dot in the upper right part). (C) MvA-Plot of the comparison of ds*Pcsut6*-injected and ds*gfp*-injected samples. *Pcsut6*’s transcript level was significantly reduced by RNAi (red dot in the lower right area). Additionally, the expression of *Pcsut25* was significantly induced (red dot in the upper right part).
(TIF)

Table S1 **Primer sets used in quantitative real-time PCR and RNAi experiments.**
(XLSX)

Table S2 **BLAST2GO results for all identified sugar transporters.** Table shows sequence description, sequence length, minimal e-value of BLAST search, mean similarity to all 5 hits for each query sequence respectively, the number of assigned gene ontology (GO) categories as well as assigned GO categories.
(XLSX)

Table S3 **Prediction of 12 transmembrane regions using TMHMM as well as Memsat2.** Furthermore, the counts of each replicate as well as sequence length and coding region’s length are listed.
(XLSX)

Table S4 **The short reads of glandular tissues were mapped onto the transcriptome using bowtie.** The short reads of the three replicates of RNA derived from the glandular tissue were mapped onto the transcriptome by applying bowtie. The percentages of aligned reads and of reads that did not align or were suppressed (due to option m) are listed. Furthermore, the numbers of reads that have been mapped are shown.
(XLSX)

Table S5 ***Pcsut*** **counts normalized to the effective library size, relatively to** ***Pcrps3*** **and** ***Pcrpl6*** **according to Livak and Schmittgen** [**71**] **.** Samples were derived from glandular tissue from juvenile *P. cochleariae*.
(XLSX)

Table S6 **Counts normalized to the effective library size of all sugar transporters after dsRNA-injection.** dsRNA targeting *Pcsut1*, *Pcsut2* and *Pcsut6* as well as dsRNA targeting *gfp* were injected. Samples were derived from glandular tissue from juvenile *P. cochleariae*.
(XLSX)
