# Supplementary figures and images for: Putative Sugar Transporters of the Mustard Leaf Beetle Phaedon cochleariae: Their Phylogeny and Role for Nutrient Supply in Larval Defensive Glands
Source: PLoS One. 2013 Dec 31;8(12):e84461. doi: 10.1371/journal.pone.0084461 (PMC3877287; doi:10.1371/journal.pone.0084461)

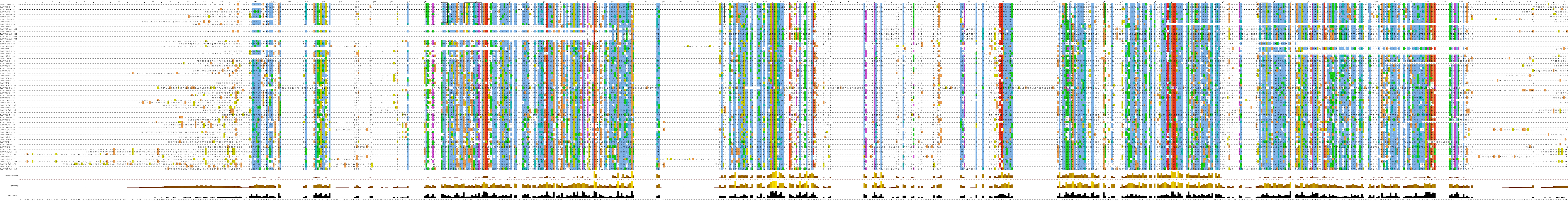

Supplement: Figure S3 — Multiple sequence alignment of the 68 chosen sugar transporters derived from P. cochleariae . The multiple sequence alignment was calculated using Probalign. The purple-branch-specific amino acids are framed. (TIF) [file pone.0084461.s003.tif]

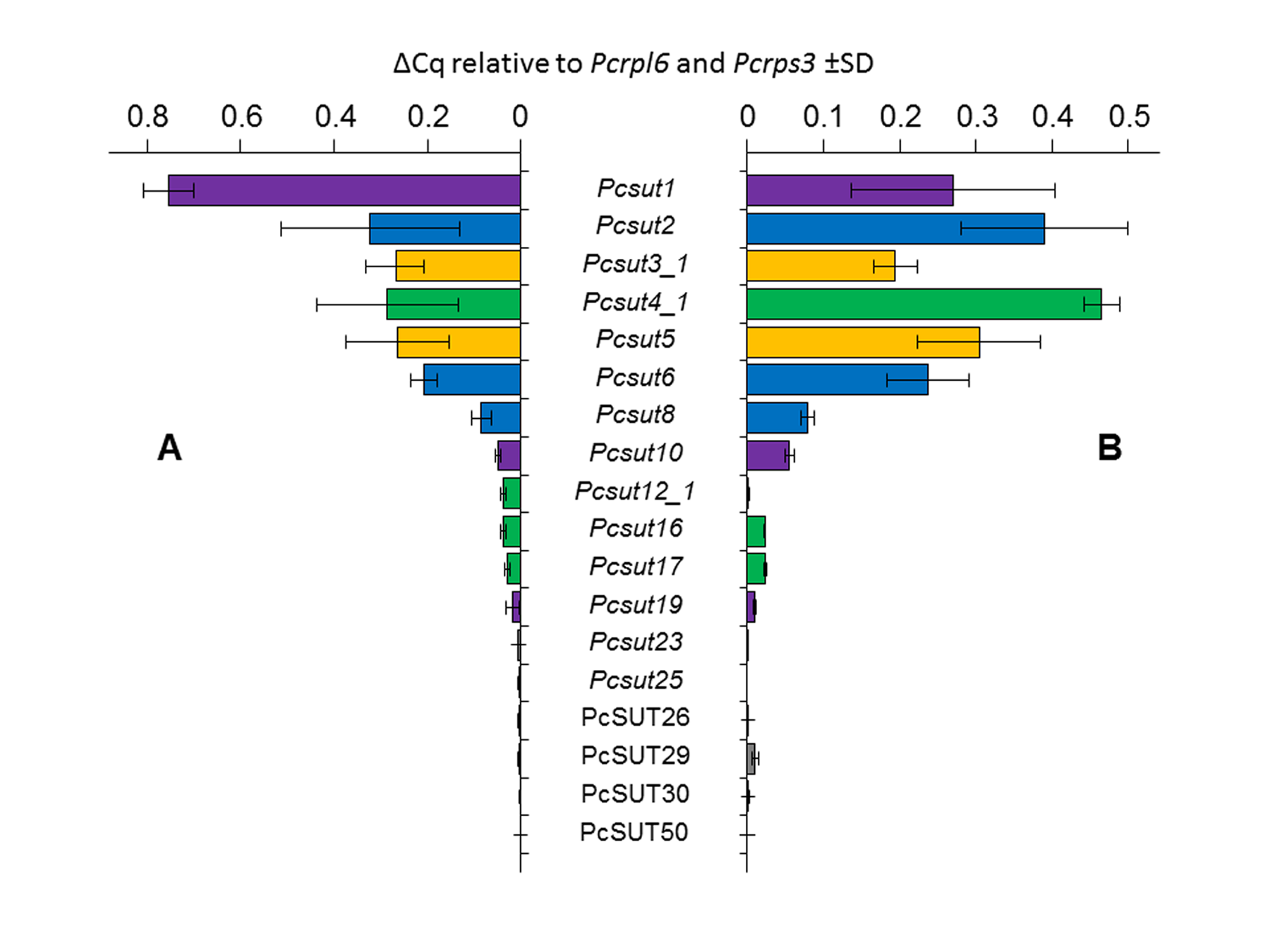

Supplement: Figure S8 — Relative mRNA levels of putative SLC2 transporters in the defensive glands of juvenile P. cochleariae determined by carrying out RNA-seq (A) and quantitative real-time PCR (B) experiments. The corresponding fold-changes of the RNA-seq samples are listed in Table S5. (TIF) [file pone.0084461.s008.tif]

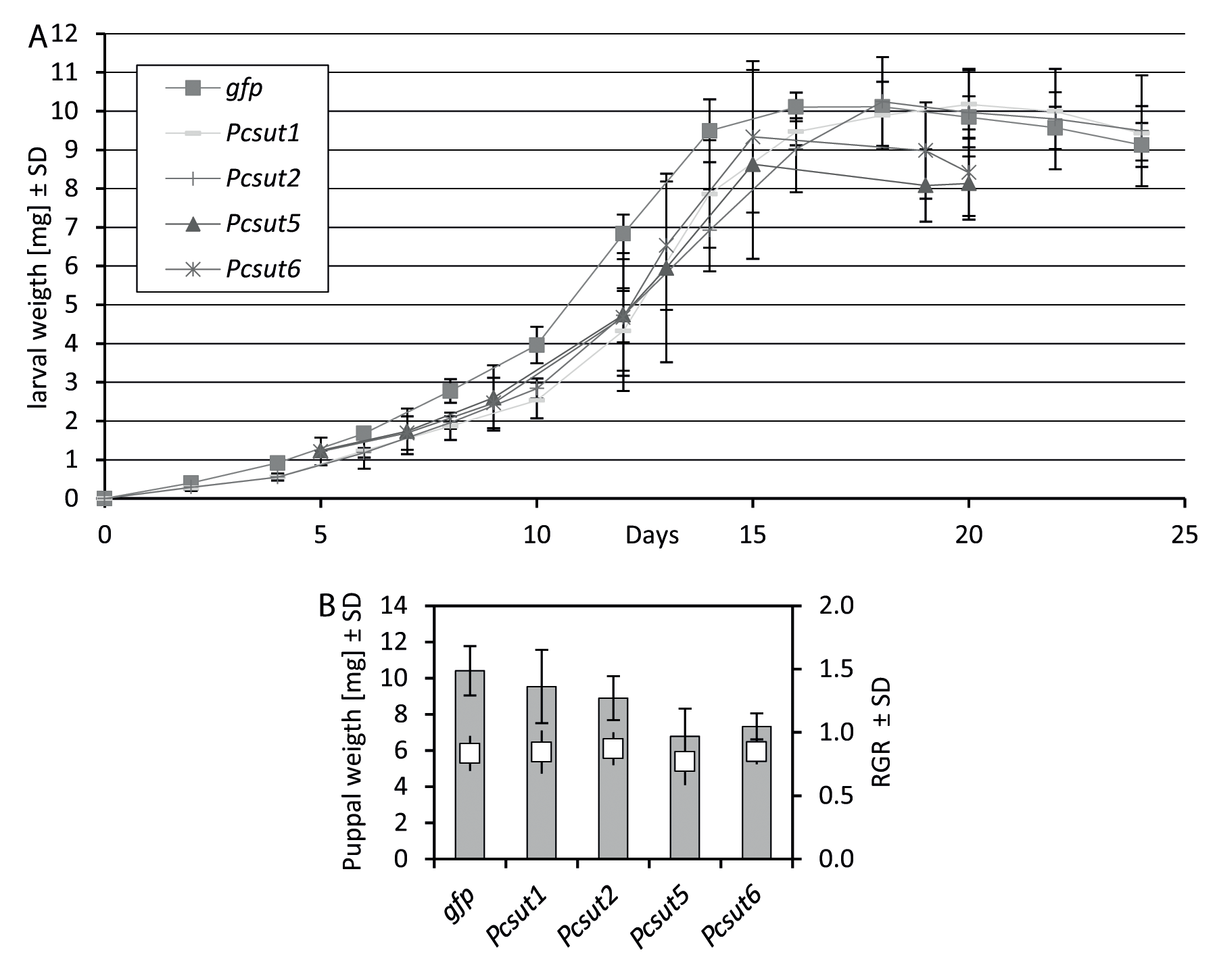

Supplement: Figure S9 — RNAi effects on the development of the larvae from P. cochleariae . A, The development of larval weight was documented and measured in a 24 or 48 h±3 h period. B, In neither the relative growth rate nor in the weight of freshly emerged pupae significant differences could be observed between dsgfp- and dsPcsut1-, dsPcsut2-, dsPcsut5-, dsPcsut6-injected larvae, n = 30. (TIF) [file pone.0084461.s009.tif]

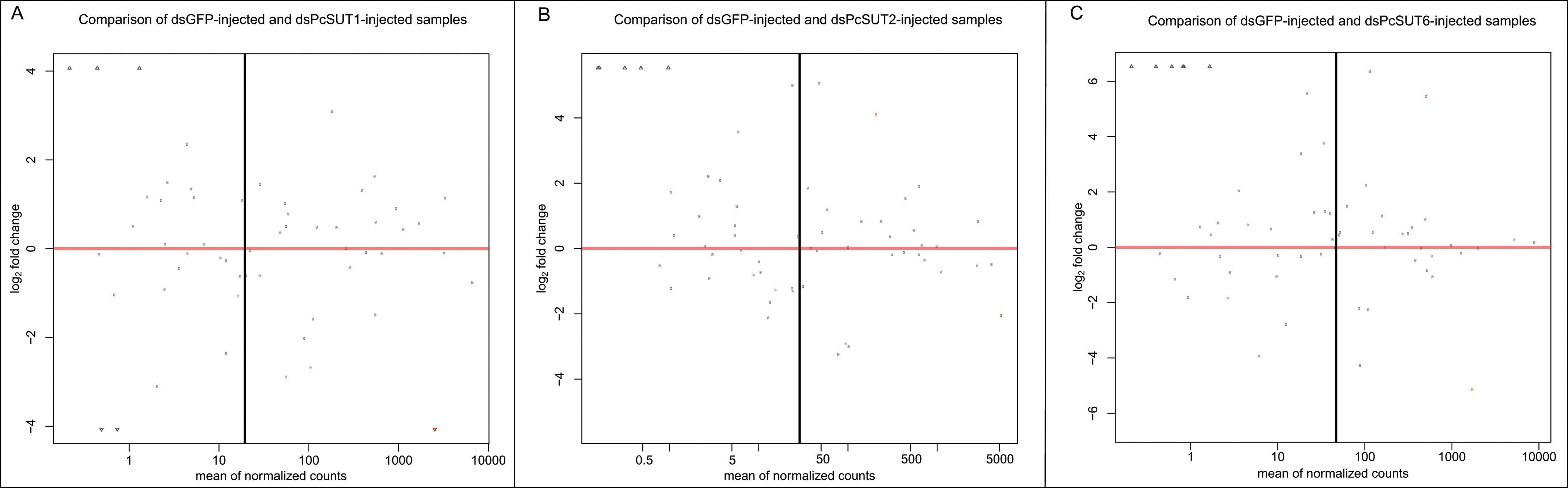

Supplement: Figure S10 — MvA-Plot showing normalized mean values versus log2fold-changes. The fold-changes (log-transformed) were computed for the comparison of dsgfp-injected and dsPcsut1-injected samples. The transcript levels are significant at 10% FDR (padj< = 0.1, padj: p-value adjusted for multiple testing with the Benjamini-Hochberg procedure which controls false discovery rate (FDR)). The differentially expressed transporters, the ones colored red, are listed in Table 4. (A) MvA-Plot of the comparison of dsPcsut1-injected and dsgfp-injected samples. Pcsut1’s transcript level was significantly reduced by RNAi (red dot in the lower right quadrant). (B) MvA-Plot of the comparison of dsPcsut2-injected and dsgfp-injected samples. Pcsut2’s transcript level was significantly reduced by RNAi (red dot in the lower right area). Additionally, the expression of Pcsut25 was significantly induced (red dot in the upper right part). (C) MvA-Plot of the comparison of dsPcsut6-injected and dsgfp-injected samples. Pcsut6’s transcript level was significantly reduced by RNAi (red dot in the lower right area). Additionally, the expression of Pcsut25 was significantly induced (red dot in the upper right part). (TIF) [file pone.0084461.s010.tif]
